# Mild oxidative stress protects against chemotherapy-induced hair loss

**DOI:** 10.3389/fonc.2022.1078916

**Published:** 2023-01-10

**Authors:** Yusheng Zhang, Joaquin J. Jimenez

**Affiliations:** Dr. Phillip Frost Department of Dermatology and Cutaneous Surgery, University of Miami Miller School of Medicine, Miami, FL, United States

**Keywords:** chemotherapy-induced hair loss, chemotherapy-induced alopecia, hair loss (alopecia), cancer, alopecia prevention, protection against hair loss, oxidative stress, cancer hair loss

## Abstract

Chemotherapy induces hair loss in most cancer patients who undergo treatment, which causes them significant psychosocial trauma. Scalp cooling has demonstrated some efficacy in attenuating chemotherapy-induced alopecia, but response rate varies between patients and chemotherapy class. Here, we showcase in rats a proof-of-concept treatment of using subcutaneous hydrogen peroxide and cumene hydroperoxide injections to provide total protection from hair loss against multiple classes of chemotherapy. We found that subcutaneous peroxides induce cell cycle arrest *via* P53 activation, thereby protecting hair follicles from the cytotoxic effects of chemotherapy on actively dividing cells. This treatment represents a highly effective and accessible way for cancer patients to maintain quality of life while undergoing treatment for cancer.

## 1 Introduction

Cytotoxic chemotherapy is still the primary treatment for many forms of cancer today. Many chemotherapeutic agents inflict irreparable DNA damage in actively-dividing cancer cells to induce cell death (1). Since 80% of scalp hair follicles are estimated to be actively growing at any moment, hair follicles are also highly susceptible to chemotherapy-induced damage (2). Most cancer patients experience chemotherapy-induced alopecia while undergoing treatment with chemotherapy. Indeed, chemotherapy-induced alopecia is unequivocally associated with cancer and causes traumatic psychosocial stress to patients, especially children (3–5). Recent clinical trials have demonstrated attenuation of chemotherapy-induced hair loss *via* scalp cooling (6–8). However, response rate varies with patients and with chemotherapy class (9, 10). To discover a better methodology preventing hair loss in cancer patients, the underlying molecular mechanisms regulating hair loss needs to be considered.

Oxidative stress is thought to augment DNA damage and worsen chemotherapy-induced alopecia (11, 12). Drugs that augment ROS (reactive oxygen species) significantly increase DNA damage-induced hair follicle apoptosis *via* activation of the P53 pathway (13, 14). Previous studies have therefore shown that P53 ablation decreases chemotherapy-induced alopecia by inhibiting apoptosis in hair follicles damaged by chemotherapy (15). However, as P53 protects against oncogenic growth, its deletion for the sake of hair loss prevention is detrimental to the larger goal of curing cancer. On the other hand, low level oxidative stress causes P53-mediated cell cycle arrest instead of apoptosis (16, 17). In line with a prior *ex vivo* study showing that G1 arrest shields hair follicles from chemotherapy, we theorized that the *in vivo* induction of P53-mediated cell cycle arrest prior to chemotherapy treatment may protect hair follicles against the cytotoxic effects of chemotherapy on actively dividing cells (18).

Here, we use an *in vivo* Rat pup model of chemotherapy-induced alopecia to demonstrate that peroxide treatment prior to chemotherapy provides near-100% protection against chemotherapy-induced alopecia. We demonstrate that peroxide-induced oxidative stress activates P53 to cause cell cycle arrest in the skin and hair follicles, thereby providing resistance to the cytotoxic effects of chemotherapy on actively dividing cells. This novel proof-of-concept treatment using commonly available reagents provides an effective solution for restoring quality of life in many cancer patients.

## 2 Materials and methods

All animal procedures were performed in compliance with the National Institutes of Health Guide for the Care and Use of Laboratory Animals and approved by the Animal Care and Use Committee of the University of Miami.

### 2.1 Pretreatment with cumene hydroperoxide and hydrogen peroxide

Long Evans (LE) Rats with nursing pups were purchased from Envigo Laboratories Inc. (8520 Allison Pointe Blvd Suite 400 Indianapolis, IN). Nursing LE rat pups were randomly assigned to receive subcutaneous injections in the nape of the neck with either vehicle of PBS (P5119-100ML, Sigma-Aldrich, St.Louis), cumene hydroperoxide (247504, Sigma-Aldrich, St. Louis, MO), or hydrogen peroxide (H1009, Sigma-Aldrich, St. Louis, MO). The cumene hydroperoxide solution was prepared at a concentration of 20 µL/mL by volume in PBS (molar concentration of 0.134 M), of which 100 µL was used per injection. Each hydrogen peroxide injection included 40 µL of the 30% stock solution and 60 µL of PBS for a total injected volume of 100 µL at a concentration of 12% by volume (molar concentration of 3.92 M). Daily injections were administered to the LE pups from day 5 until day 10 after birth, following which chemotherapy administration was started.

### 2.2 Combination treatment with low-dose cumene hydroperoxide and RITA

Nursing LE rat pups were randomly assigned to receive subcutaneous injections in the nape of the neck with either PBS, low-dose cumene hydroperoxide, the P53 activator RITA (Reactivation of p53 and Induction of Tumor cell Apoptosis) (S2781, Selleck Chemicals, Houston, TX), or a combination of low-dose cumene hydroperoxide and RITA. Low-dose cumene hydroperoxide was prepared at a concentration of 4 µL/mL in PBS, of which 50 µL was administered with each injection for a 10-fold decrease in cumene hydroperoxide used. RITA was initially solubilized in DMSO in accordance with manufacturer suggestions and diluted to a final concentration of 40 µg/mL in PBS, of which 50 µL was administered with each injection. The rats receiving the combination of low-dose cumene hydroperoxide and RITA received both the 50 µL injection of cumene hydroperoxide and the 50 µL injection of RITA for a final total volume of 100 µL. Daily injections were administered to the LE pups from day 5 until day 10 after birth along the same timeline as previously discussed, following which chemotherapy administration was started.

### 2.3 Treatment with chemotherapy

Following pre-treatment with either PBS, cumene hydroperoxide, or hydrogen peroxide, the LE rat pups were then given chemotherapy intraperitoneally starting at 11 days of age. Stock solutions of VP-16 (Etoposide, 1268808, Sigma-Aldrich, St. Louis MO, USA) was prepared at a concentration of 20 mg/ml in 30.5% ethanol in H_2_O supplemented with 2 mg/ml citric acid, 30 mg/ml benzyl alcohol, 80 mg/ml Tween-80, and 150 mg/ml PEG-300. VP-16 stock solution was diluted in PBS and was given intraperitoneally at a concentration of 1.5 mg/kg for three days. Cytoxan (cyclophosphamide, PHR1404, Sigma-Aldrich, St. Louis MO, USA) was prepared in H_2_O supplemented with 5% dextran and was also diluted appropriately in PBS. Cytoxan and was given at a concentration of 35 mg/kg for one day only. The rat pups that received the combination of Cytoxan and Adriamycin (Doxorubicin, 1225703, Sigma-Aldrich, St. Louis MO, USA) were first administered with Cytoxan at a concentration of 25 mg/kg for one day followed by three days of Adriamycin that was prepared in H_2_O and given at a concentration of 1.5 mg/kg. Alopecia was recorded seven days after the last injection of chemotherapy. Since all rats pre-treated with either cumene hydroperoxide or hydrogen peroxide exhibited 100% or near 100% protection from hair loss, data were displayed in the figures by either (+) or (–) for whether any hair was protected at the site of injection.

### 2.4 Histological analyses

Skin at the site of vehicle and cumene hydroperoxide injections were isolated from the nape of rats on day 20 after birth (day 7 after treatment with VP-16, day 9 after treatment with Cytoxan, day 6 after treatment with Cytoxan-Adriamycin). Histological analyses were performed following our previously-described protocol (19). Briefly, Paraffin sections were stained with hematoxylin and eosin, and the stained slides were subsequently evaluated using an Axio Observer D1 microscope and AxioVision software (Carl Zeiss Microimaging, Thornwood, NY). Images were captured using a Keyence Biozero BZ–800 K Microscope and analyzed using Image J image analysis software.

### 2.5 RNA-seq analyses

Samples of rat skin were cleared of hair and debris prior to submission to Ocean Ridge Biosciences (array@oceanridgebio.com) for mRNA-Sequencing. According to the vendor provided protocol, total RNA was isolated using the TRI Reagent (Molecular Research Center; Part # TR118) method. After isolation, RNA was quantified by O.D. measurement and assessed for quality on a 1% agarose – 2% formaldehyde RNA Quality Control (QC) gel. The RNA was then digested with RNase free DNase I (Epicentre; Part # D9905K) and re-purified using Agencourt RNAClean XP beads (Beckman Coulter; Part # A63987). The newly digested RNA samples were then quantified by O.D. measurement. Amplified cDNA libraries suitable for sequencing were prepared from 250 nanograms (ng) of DNA-free total RNA using the TruSeq Stranded mRNA Library Prep (Illumina Inc.; Part # 20020595). The quality and size distribution of the amplified libraries were determined by chip-based capillary electrophoresis (Bioanalyzer 2100, Agilent Technologies). Libraries were quantified using the KAPA Library Quantification Kit (Kapa Biosystems, Boston, MA). Libraries were pooled at equimolar concentrations and sequenced on the Illumina NextSeq 500 sequencer using one Mid Output v2 150 cycle kit (part# FC-404-2001). In each case the libraries were sequenced with 76 nt paired-end reads plus 8 nt dual-index reads on the instrument running NextSeq Control Software version 2.2.0.4. Reads were generated into FASTQ files using the default setting on Illumina’s bcl2fastq program v2.17.1.14 and were filtered and trimmed. The reads were aligned to the hg38 genome using HISAT2 version 2.0.5 along the following parameters (N = 1, L = 20, i = S,1,0.5). Read count was performed using the featureCounts program version 1.5.1 for gene-level counting (http://subread.sourceforge.net). Normalized RPKM values were calculated from the raw featureCounts read counts using the formula (Gr x 1,000,000)/(Gl x mRNArt) where Gr is raw read count for a gene, Gl is length of the exon model for the gene, and mRNArt is the total read count for exons from nuclear-derived protein-coding mRNAs. The RPKM values were further filtered to retain a list of genes with a minimum of 50 mapped reads in all 3 samples to ensure maximum technical reproducibility. Differential expression analysis was performed in R studio (R version 3.6.3) using the DESeq2 package with a q-value < 0.05 and a fold change > 1.5. Heatmap analysis was also performed in R studio using the pheatmap package (version 1.0.12) along parameters that specify two distinct clusters. Gene ontology and pathway analyses were performed using EnrichR (20–22). Raw RNA-seq data is provided in **Table S1**, and processed and filtered RNA-seq data is provided in **Table S2**.

## 3 Results

### 3.1 Subcutaneous peroxide treatment prior to initiating chemotherapy prevents chemotherapy-induced alopecia

**Figure 1:**
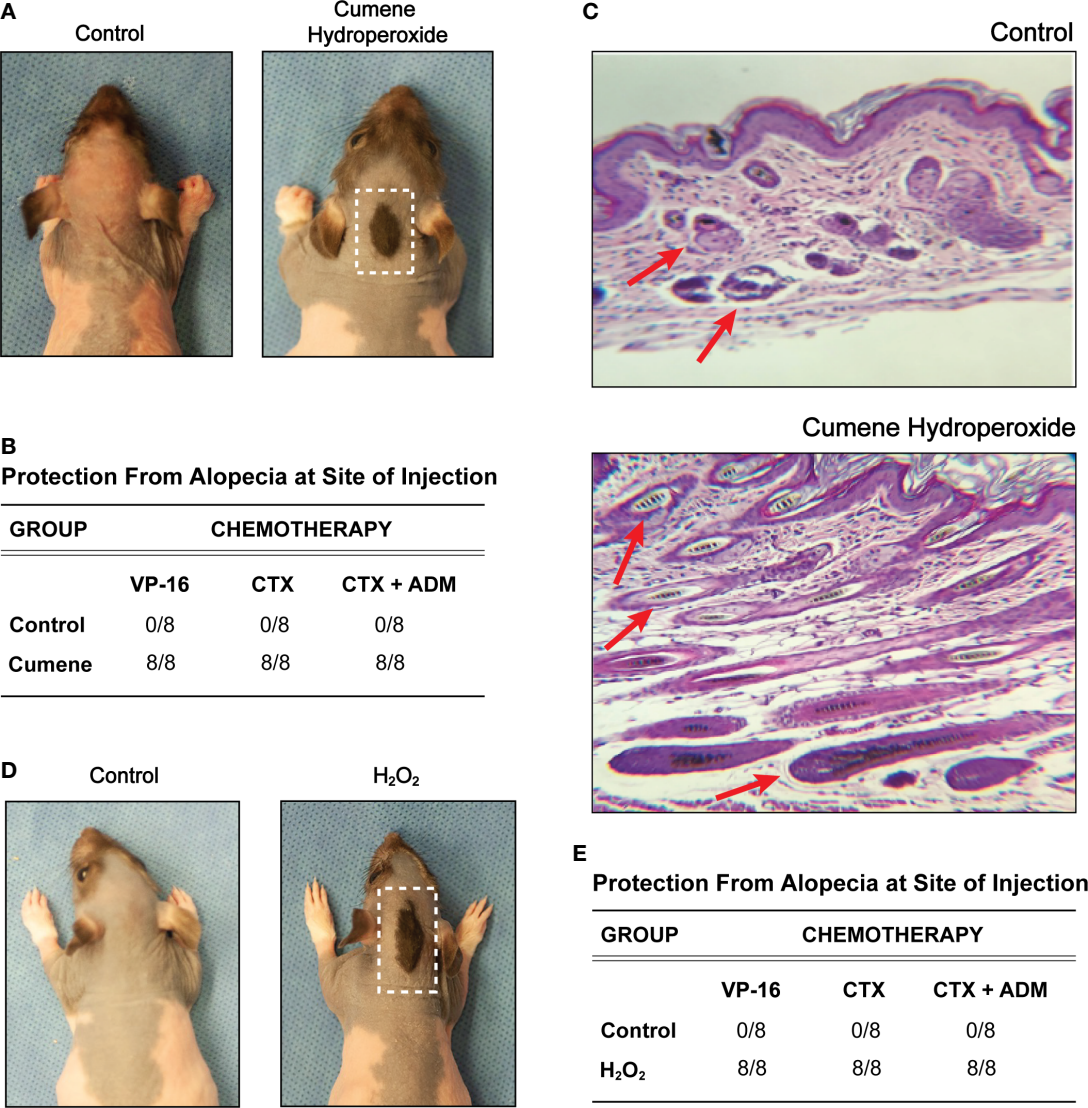
Pre-treatment with cumene hydroperoxide prevents chemotherapy-induced alopecia. **(A)** Representative photographic images of VP16-induced alopecia in control rats (left) and rats pre-treated with cumene hydroperoxide. The scalp area with a preserved coat of hair demarks the site of cumene hydroperoxide injection and is outline in white. **(B)** Table compilation of alopecia protection at the injection site in control rats and rats pre-treated with cumene hydroperoxide after completing a course of Etoposide (VP-16), Cytoxan (CTX), or Cytoxan-Adriamycin (CTX-ADM) treatment. N = 8 per treatment group. **(C)** Representative light microscopic sections of skin biopsy obtained from control rats and cumene hydroperoxide treated rats (bottom) seven days post chemotherapy. Slides were stained with hemotoxylin and eosin. Red arrows denote representative hair follicles that are damaged (above) and healthy (below). **(D)** Representative photographic images of VP16-induced alopecia in control rats (left) and rats pre-treated with hydrogen peroxide (H_2_O_2_). The scalp area with a preserved coat of hair demarks the site of H_2_O_2_ injection and is outlined in white. **(E)** Table compilation of alopecia protection at the injection site in control rats and rats pre-treated with H_2_O_2_ after completing a course of Etoposide (VP-16), Cytoxan (CTX), or Cytoxan-Adriamycin (CTX-ADM) treatment. N = 8 per treatment group.

Cumene hydroperoxide is known to exert oxidative stress by inducing intracellular alkyl radicals in murine keratinocytes, and cumene hydroperoxide has been widely used to model oxidative stress *in vivo* (23–26). Fortunately, studies have shown that short term (< 14 days) subcutaneous administration of cumene hydroperoxide produces minimal cutaneous irritation in rats (26), making cumene hydroperoxide an ideal choice for exogenously introducing cutaneous oxidative stress. Using the previously described and widely used rat pup model of chemotherapy-induced alopecia (27), we administered daily subcutaneous injections of either cumene hydroperoxide or PBS (phosphate-buffered saline) into the nape of Long Evans rat pups for six days, followed by daily intraperitoneal injections of the chemotherapeutic agent VP-16 (etoposide) for three days. Upon seven days after the completion of VP-16 injections, all rats pre-treated with cumene hydroperoxide maintained a coat of hair at the site of cumene hydroperoxide injection whereas all rats pre-treated with vehicle did not (**Figures 1A, B**). Cumene hydroperoxide provided similar hair loss protection against monotherapy with CTX (cyclophosphamide) and combination therapy with CTX-ADM (Cyclophosphamide-Doxorubicin) (**Figure 1B**). This demonstrates that pre-treatment with subcutaneous cumene hydroperoxide prior to the start of chemotherapy prevents alopecia induced by multiple classes of chemotherapeutic agents. Histological analyses of skin biopsies taken from the site of cumene hydroperoxide and vehicle injections show that VP-16 induced extensive hair follicle damage and dispersion of melanin in the skin of rats pre-treated with vehicle only (**Figure 1C**). However, the skin of rats pre-treated with cumene hydroperoxide showed minimal to no hair follicle damage nor melanin dispersion following VP-16 treatment (**Figure 1C**). Additionally, the skins of cumene hydroperoxide pre-treated rats appeared to be thicker than that of vehicle pre-treated rats, although objective measurements were not obtained. This lends further evidence to suggest that pre-treatment with subcutaneous cumene hydroperoxide protects against the cytotoxic effects of chemotherapies such as VP-16.

Similar to cumene hydroperoxide, H_2_O_2_ (hydrogen peroxide) is a ROS that is known to generate oxidative stress and has been clinically used in numerous cutaneous applications (28). However, in contrast to cumene hydroperoxide, H_2_O_2_ is produced endogenously in most living organisms, and low-cost commercial H_2_O_2_ solutions are widely available. We therefore performed the same set of experiments using subcutaneous H_2_O_2_ injections. Like cumene hydroperoxide, pre-treatment with daily H_2_O_2_ injections for six days also provided full protection against alopecia induced by VP-16 as well as by CTX monotherapy and CTX-ADM combination therapy at the site of injection (**Figures 1D, E**). Of note, we initially intended to score the alopecia on a scale of 0 to 4 based on the degree of hair loss. However, since all rats pre-treated with either cumene hydroperoxide or H_2_O_2_ exhibited 100% or near 100% protection from hair loss, data were displayed in the figures by either (+) or (–) for whether any hair was protected at the site of injection. These results therefore show that pre-treatment with ROS-producing compounds can protect hair follicles against the cytotoxic effects of chemotherapy and prevent chemotherapy-induced alopecia.

### 3.2 Cumene hydroperoxide activates P53-mediated cell cycle arrest to prevent chemotherapy-induced alopecia

**Figure 2:**
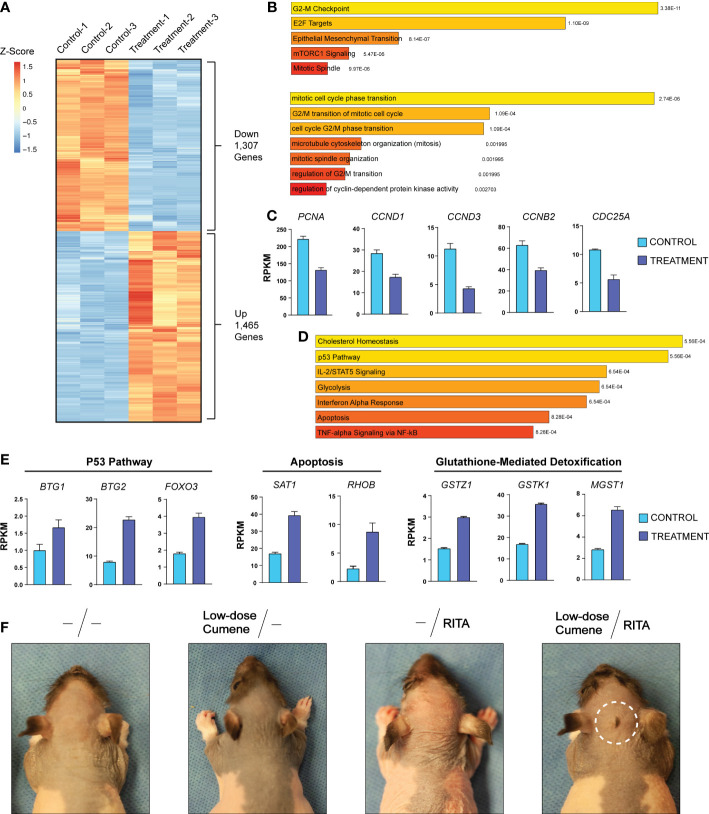
Cumene Hydroperoxide Protects Against Chemotherapy-Induced Alopecia by Activating the P53 Pathway to Arrest Cell Cycle. **(A)** RNA-seq heat maps of all differentially expressed genes in control and cumene hydroperoxide-treated rat skin. 1,307 genes and 1,406 genes were found to be significantly down- and upregulated, respectively. Fold change > 1.5, FDR < 0.05, N=3. **(B)** MSigDB Hallmark (Molecular Signature Database Hallmark) gene enrichment analysis and Gene Ontology of Biological Processes (bottom) of genes downregulated upon cumene hydroperoxide treatment. **(C)** Bar graphs depicting the expression levels of specific genes important for stimulating cell cycle progression in control and treated samples. Error bars represent the standard deviation of 3 independent replicates. **(D)** MSigDB Hallmark gene enrichment analysis of genes upregulated upon cumene hydroperoxide treatment. **(E)** Bar graph depicting the expression levels of specific genes implicated in the P53 Pathway (left), Apoptosis (middle), and Glutathione-mediated Detoxification (right) in control and treated samples. Error bars represent the standard deviation of 3 independent replicates. **(F)** Representative photographic images of VP16-induced alopecia in rats that were given no pre-treatment, pre-treatment with low-dose cumene hydroperoxide, with RITA, or with both RITA and low-dose cumene hydroperoxide. The scalp area with a preserved coat of hair is outlined in white. N = 6 per treatment group.

To further investigate the molecular mechanisms underlying how cumene hydroperoxide protects against chemotherapy-induced alopecia, we performed total RNA-seq using skin harvested from the injection sites of vehicle as well as cumene hydroperoxide-treated rats upon completing the six days of cumene hydroperoxide injections. We found that 1,307 genes and 1,465 genes were significantly down- and upregulated, respectively, in the skin of cumene hydroperoxide-treated rats when compared to that of the vehicle-treated rats (**Figure 2A**). Gene enrichment analysis showed that E2F Target genes as well as genes that regulate the G2-M checkpoint were significantly downregulated (**Figure 2B** – top). Additionally, gene ontology analysis shows downregulation of processes such as mitotic cell cycle phase transition and G2/M transition of mitotic cell cycle (**Figure 2B** – bottom). Specific genes important for cell cycle progression and cellular division such as *PCNA* (proliferating cell nuclear antigen), *CCND1* (cyclin D1), and *CCND3* (cyclin D3) were also significantly downregulated by cumene hydroperoxide treatment (**Figure 2C**). This therefore shows that treatment with cumene hydroperoxide causes cell cycle arrest in the skin and hair follicles of cumene hydroperoxide-treated rats. On the other hand, gene enrichment analysis of the 1,465 upregulated genes showed significant upregulation in genes implicated in the P53 pathway as well as P53-regulated processes such as Apoptosis (**Figure 2D**). Genes involved in inflammation-related pathways such as cholesterol homeostasis and IL-2/STAT5 signaling were also significantly upregulated (**Figure 2D**). Specific target genes of the P53 pathway (i.e. *BTG1*, *BTG2*, *FOXO3*) and the apoptotic pathway (*SAT1*, *RHOB*) were significantly upregulated by cumene hydroperoxide treatment (**Figure 2E**). Interestingly, genes involved in glutathione-mediated detoxification pathway such as *GSTZ1*, *GSTK1*, and *MGST1* were also significantly upregulated (**Figure 2E**). Since glutathione is one of the main mechanisms of protection against ROS in mammalian cells (29), its upregulation indicates a coordinated protective response against cumene hydroperoxide-induced inflammation and oxidative stress in the rat skin and hair follicles. This therefore suggests that cumene hydroperoxide-induced oxidative stress produces inflammation that leads to P53-mediated cell cycle arrest in rat skin and hair follicles.

To further investigate the link between cumene hydroperoxide, P53 pathway activation, and cell cycle arrest, we decided to test whether treatment with the P53 activator RITA (Reactivation of p53 and Induction of Tumor cell Apoptosis) can potentiate the protective effects of cumene hydroperoxide (30, 31). We therefore pre-treated four groups of six rats with daily subcutaneous injections of either vehicle, low-dose cumene hydroperoxide, RITA (140 µM), or a combination of low-dose cumene hydroperoxide and RITA for six days prior to chemotherapy with VP-16. We found that VP-16 induced total alopecia in all rats pre-treated with either vehicle, low-dose cumene hydroperoxide, or RITA (**Figure 2F**). However, only the combination of RITA and low-dose cumene hydroperoxide preserved a patch of hair at the site of injection in all six treated rats (**Figure 2F**). Although the protected area of hair is smaller than that of therapeutic levels of cumene hydroperoxide (**Figure 1A**), this result nevertheless shows that co-treatment with the P53-activating RITA enhances the protective effects of low-dose cumene hydroperoxide that is ineffective at preventing alopecia when given alone. This result thereby provides further evidence to suggest that cumene hydroperoxide protects against chemotherapy-induced alopecia by inducing P53-mediated cell cycle arrest in hair follicles.

## 4 Discussions

Chemotherapy-induced alopecia and the appearance of severe hair loss is synonymous with cancer. For many undergoing treatment for cancer, hair loss inflicts significant trauma and represents a major barrier to living a life with normalcy. Scalp cooling therapy has recently been shown to reduce hair loss in patients receiving chemotherapy. However, its efficacy varies between patients and with different classes of chemotherapy, and a large proportion of patients may still lose up to 50% of their hair. Here, using the rat pup model of chemotherapy-induced alopecia, we showcase a novel proof-of-concept treatment that entirely prevents hair loss induced by many different classes of chemotherapy. This model has been characterized and validated by previously published studies (27, 32). Furthermore, the treatment is based on inexpensive peroxides such as cumene hydroperoxide and hydrogen peroxide, the latter being an endogenously occurring and widely available solution found at local pharmacies across the world. This would therefore remove major barriers for cancer patients to receive treatment for chemotherapy-induced alopecia and would ensure a more equitable access to cancer medical care.

Besides our study here, only one other previously published study by Botchkarev et al. has demonstrated similar levels of *in vivo* prevention of chemotherapy-induced alopecia (15). However, this was accomplished by deleting P53, which is an important tumor suppressor whose ablation is detrimental to the curative goal of chemotherapy. Another recent study by Purba et al. used the CDK4/6 inhibitor Palbociclib in an *ex vivo* model to induce a G1 cell cycle arrest in hair follicle stem cells, thereby protecting this niche of cells from taxane-induced cytotoxic damages (18). This however has not yet been demonstrated in an *in vivo* model, and the price of Palbociclib would be cost-prohibitive for many patients already undergoing expensive cancer treatments. Here, we used low-cost cumene hydroperoxide and hydrogen peroxide to generate P53-activating oxidative stress to induce cell cycle arrest, therefore protecting hair follicles from the damaging effects of chemotherapy on actively dividing cells. A recent study by Chen et al. discovered that scalp cooling protects against chemotherapy-induced alopecia partially *via* slowing down the cell cycle in hair follicles, making hair follicles resistant to the cytotoxic effects of chemotherapy (33). This therefore serves as an additional line of evidence to show that cell cycle arrest renders hair follicles resistant to cytotoxic chemotherapy.

One major drawback of this treatment is that cumene hydroperoxide and/or hydrogen peroxide would be injected subcutaneously into the scalp, and neither peroxides have yet been characterized in any therapeutic capacity. However, a report from 2017 reported a case of oxygen embolism produced by accidental introduction of subcutaneous hydrogen peroxide (34). This suggests that caution should be taken if subcutaneous hydrogen peroxide injections were to be explored further as a modality of therapy. Nevertheless, based on our observation, no major side effects were observed upon completing the subcutaneous hydrogen peroxide injections. Recent clinical trials have also shown that low-level pain and discomfort are some of the most commonly reported side effects associated with subcutaneous injections (35). Therefore, it may be beneficial for patients to use a topical peroxide preparation. Future work may explore ways to formulate topical peroxide preparations that can deliver high doses of oxidants subcutaneously. Additionally, existing topical peroxide formulations such as benzoyl peroxide may be explored for its potential in preventing chemotherapy-induced alopecia.

## Data availability statement

The datasets presented in this study can be found in online repositories. The names of the repository/repositories and accession number(s) can be found in the article/**Supplementary material**.

## Ethics statement

The animal study was reviewed and approved by University of Miami Institutional Animal Care and Use Committee.

## Author contributions

JJ conceived the concept of the treatment. JJ designed the experiments with contributions from YZ and JJ performed the experiments and YZ performed the analyses. YZ made the figures and wrote the original draft of the paper. JJ and YZ both contributed to the review and editing. All authors contributed to the article and approved the submitted version.

## Supplementary material

Supplementary Table 1 Raw RNA-seq data.

Supplementary Table 2 Processed and Filtered RNA-seq data.
